# Remimazolam–Propofol and Propofol Induction Regimens for Pediatric Bidirectional Endoscopy: A Three-Arm Randomized Trial

**DOI:** 10.3390/children13081056

**Published:** 2026-08-08

**Authors:** Liming Cai, Zhengzheng Gao, Zeyang Wang, Xiaoxue Wang, Jing Hu, Lei Hua, Wenya Fu, Lanxin Qiao, Jianmin Zhang, Lijing Li, Fang Wang, Xiumin Qin

**Affiliations:** 1 Department of Anesthesiology, National Center for Children’s Health, Beijing Children’s Hospital, Capital Medical University, Beijing 100045, China; cailiming19931008@126.com (L.C.); sunbrightangel@sina.com (Z.G.); wzy1996126@126.com (Z.W.); wnagxiangshu1215@163.com (X.W.); doctor_hj@icloud.com (J.H.); orangehua@sina.com (L.H.); fuwenya@163.com (W.F.); qiaolanxin@126.com (L.Q.); zhangjianmin@bch.com.cn (J.Z.); amber1717@163.com (L.L.); 2 Department of Gastroenterology, Digestive Endoscopy Center, National Center for Children’s Health, Beijing Children’s Hospital, Capital Medical University, Beijing 100045, China; qinxiumin0418@126.com

**Keywords:** remimazolam, propofol, pediatric anesthesia, gastrointestinal endoscopy, respiratory depression

## Abstract

**Highlights:**

**What are the main findings?**

- Both remimazolam–propofol induction regimens reduced monitor-defined respiratory depression compared with propofol 3 mg/kg.
- In an exploratory comparison, the 0.2 mg/kg remimazolam regimen had a shorter time to eye opening than the 0.4 mg/kg regimen.

**What are the implications of the main findings?**

- These findings support further evaluation of remimazolam–propofol as a regimen strategy for pediatric bidirectional endoscopy.
- Trials that record event-linked airway interventions are needed before selecting a preferred regimen.

**Abstract:**

**Background/Objectives:** Propofol is effective for pediatric endoscopy but can depress ventilation. We compared three induction regimens in school-aged children undergoing bidirectional gastrointestinal endoscopy. **Methods:** This single-center, assessor-blinded randomized trial allocated children aged 6–12 years (1:1:1) to propofol 3 mg/kg (P), remimazolam 0.2 mg/kg plus propofol 1 mg/kg (R1), or remimazolam 0.4 mg/kg plus propofol 1 mg/kg (R2). All groups received sufentanil and standardized propofol maintenance. Respiratory depression was a respiratory rate < 10 breaths/min or peripheral oxygen saturation < 90% for >1 min. The trial was prospectively registered (ChiCTR2500096218), and analysis followed assigned groups. **Results:** The analysis included 120 children (40 per group). Respiratory depression occurred in 32/40 (80.0%) in P, 6/40 (15.0%) in R1, and 14/40 (35.0%) in R2 (overall *p* < 0.001). Compared with P, the risk difference was −65.0 percentage points for R1 (95% confidence interval [CI], −77.4 to −44.6; risk ratio, 0.19; 95% CI, 0.09–0.37) and −45.0 percentage points for R2 (95% CI, −61.0 to −23.6; risk ratio, 0.44; 95% CI, 0.27–0.66); both Holm-adjusted *p* values were <0.001. Mean time to eye opening was 12.7, 11.2, and 16.9 min, respectively. In the exploratory R1-versus-R2 comparison, time to eye opening was 5.75 min shorter with R1 (95% CI, 4.28–7.20). **Conclusions:** Both remimazolam–propofol regimens reduced monitor-defined respiratory depression compared with propofol 3 mg/kg. The 0.2 mg/kg remimazolam–propofol regimen combined this respiratory benefit with faster recovery than the 0.4 mg/kg regimen in exploratory analysis, supporting its further evaluation as an induction strategy for pediatric bidirectional endoscopy.

## 1. Introduction

Same-session esophagogastroduodenoscopy and colonoscopy can avoid repeated bowel preparation and anesthesia exposure in children who require evaluation of both the upper and lower gastrointestinal tract. These procedures require immobility, adequate depth of anesthesia, and continuous cardiorespiratory monitoring. Propofol has a rapid onset and short recovery, but its narrow therapeutic range and dose-dependent respiratory effects remain relevant during nonintubated pediatric endoscopy [1].

Remimazolam is an ultra-short-acting benzodiazepine metabolized by tissue esterases [2,3,4]. Randomized adult trials have generally reported fewer respiratory or hemodynamic events with remimazolam than with propofol, although protocols, opioid co-administration, outcome definitions, and procedures differ [5,6,7,8,9,10]. Systematic reviews also suggest a lower frequency of respiratory depression with remimazolam, but the certainty and magnitude of the effect vary across procedural settings [11,12,13,14,15,16,17]. Combination studies in which remimazolam was added while the propofol dose was reduced evaluate the complete regimen rather than the independent effect of either drug [9,10].

Evidence in children is more limited. A randomized trial of pediatric bidirectional endoscopy compared remimazolam and propofol, each combined with low-dose esketamine, and reported fewer respiratory events in the remimazolam group [18]. Remimazolam has also been studied in pediatric general anesthesia and magnetic resonance imaging [19,20]. A three-dose pediatric gastroscopy trial compared remimazolam combined with sufentanil and titrated propofol, with propofol consumption as the primary outcome [21]. A recent pediatric review found that available studies remain heterogeneous in procedure type, co-medication, and outcome definitions [22].

We compared three induction regimens in children undergoing bidirectional endoscopy under deep intravenous anesthesia without planned airway instrumentation: propofol 3 mg/kg, remimazolam 0.2 mg/kg plus propofol 1 mg/kg, and remimazolam 0.4 mg/kg plus propofol 1 mg/kg. The primary objective was to compare respiratory depression. Secondary objectives were to compare cardiovascular depression, supplemental propofol use, procedure duration, recovery time, sedation success, and other adverse events. We hypothesized that each remimazolam–propofol regimen would reduce respiratory depression relative to propofol 3 mg/kg.

## 2. Materials and Methods

### 2.1. Study Design, Setting, and Ethics

This prospective, single-center, assessor-blinded, three-arm parallel randomized controlled trial was conducted at the Digestive Endoscopy Center of Beijing Children’s Hospital, Capital Medical University, Beijing, China. Recruitment occurred from 5 February to 31 August 2025. Follow-up continued through discharge from the post-anesthesia care unit. The trial used a superiority framework and a 1:1:1 allocation ratio.

The Ethics Committee of Beijing Children’s Hospital, Capital Medical University approved the protocol on 26 November 2024 (approval No. [2024-Y-278-D). A parent or legal guardian provided written informed consent before enrollment. The trial was prospectively registered in the Chinese Clinical Trial Registry on 20 January 2025 (ChiCTR2500096218), before enrollment of the first participant. This report follows CONSORT 2025 [23].

### 2.2. Participants

Children were eligible if they were 6–12 years old, had American Society of Anesthesiologists physical status I or II, were scheduled for combined diagnostic esophagogastroduodenoscopy and colonoscopy under deep intravenous anesthesia without planned tracheal intubation, had a body mass index no greater than 24.0 kg/m^2^, and had written parental or guardian consent.

Pre-randomization exclusion criteria were an emergency procedure; severe cardiac, pulmonary, or other major-organ dysfunction; a planned therapeutic endoscopic procedure, including polypectomy or endoscopic dilation; anticipated procedure duration longer than 60 min; severe obstructive sleep apnea, defined as an obstructive apnea–hypopnea index above 10 events/h; an anticipated difficult airway; high aspiration risk, including active upper gastrointestinal bleeding; allergy or hypersensitivity to a study drug; or participation in another drug trial during the preceding 3 months. A change from diagnostic to therapeutic endoscopy after randomization was not an analysis exclusion. These children remained in their assigned groups.

### 2.3. Randomization and Blinding

An independent researcher who was not involved in participant recruitment, anesthesia administration, or outcome assessment generated a computer-based random-number table and assigned participants in a 1:1:1 ratio to the propofol group (P), lower-dose remimazolam–propofol group (R1), or higher-dose remimazolam–propofol group (R2). Group assignments were placed in consecutively numbered, opaque, sealed envelopes and kept by another researcher who was not involved in anesthesia administration or outcome assessment. After eligibility had been confirmed and written informed consent obtained from a parent or legal guardian, the next envelope was opened in enrollment order. The treating anesthesiologist then learned the assignment and administered the allocated regimen. Personnel responsible for intraoperative monitoring and outcome assessment did not participate in allocation, had no access to the allocation sequence or unopened envelopes, and remained blinded to group assignment.

The anesthesiologist administering the study drugs was not blinded because the regimens required different preparations and administration sequences. Children and their parents or guardians were unaware of group assignment. A designated investigator who did not provide anesthesia and was unaware of treatment allocation observed the monitor and recorded outcomes. The endoscopist was not involved in allocation or outcome recording.

### 2.4. Periprocedural Management and Interventions

Children fasted for at least 6 h after solid food or formula, 4 h after breast milk, and 2 h after clear liquids. Peripheral intravenous access was established before transfer to the endoscopy suite. The same anesthesiologist delivered all study regimens.

Continuous electrocardiography, pulse oximetry, respiratory rate monitoring, and noninvasive blood pressure monitoring (Shenzhen Mindray Bio-Medical Electronics Co., Ltd., Shenzhen, China) began on arrival. Prespecified values were transcribed at 5 min intervals, while the monitor display was observed continuously for outcome ascertainment. Respiratory rate was measured by capnography: exhaled gas was continuously sampled through a carbon dioxide sampling line connected to the nasal oxygen cannula, and the monitor calculated and displayed respiratory rate from the end-tidal carbon dioxide waveform. The blinded observer used the displayed respiratory rate when the waveform was continuous, regular, and interpretable. The same monitoring equipment and sampling setup were used in all groups. Supplemental oxygen was administered by nasal cannula at 2 L/min before induction.

All children received sufentanil 0.1 µg/kg (Yichang Humanwell Pharmaceutical Co., Ltd., Yichang, China). P received propofol 3 mg/kg (Xi’an Libang Pharmaceutical Co., Ltd., Xi’an, China). R1 received remimazolam 0.2 mg/kg (Jiangsu Hengrui Pharmaceuticals Co., Ltd., Lianyungang, China) followed by propofol 1 mg/kg. R2 received remimazolam 0.4 mg/kg followed by propofol 1 mg/kg. Sufentanil was administered first, followed by remimazolam in R1 and R2 and then propofol. Each drug was administered over at least 30 s, with at least 1 min between successive drugs.

Gastroscope insertion was attempted when the Modified Observer’s Assessment of Alertness/Sedation score was 1 or lower. If the score remained above 1 at 2 min after induction or movement occurred during insertion, propofol 1 mg/kg was administered. The bolus could be repeated at intervals of at least 2 min until adequate insertion conditions were achieved. Anesthesia was maintained with propofol 4 mg/kg/h. Movement or poor tolerance during the procedure prompted an additional propofol bolus of 1 mg/kg.

If peripheral oxygen saturation fell below 90%, the airway was opened with a jaw lift. If saturation did not improve, nasal oxygen flow was increased to 6 L/min. Persistent hypoxemia prompted interruption of the procedure, withdrawal of the endoscope, and mask positive-pressure ventilation. A laryngeal mask airway or tracheal tube was used if ventilation remained inadequate. Hypotension or bradycardia was treated with intravenous fluid or vasoactive medication at the anesthesiologist’s discretion.

The maintenance infusion was stopped when the colonoscope reached the ileocecal valve. Children were transferred to the post-anesthesia care unit for monitoring and oxygen supplementation and returned to the ward after achieving a modified Aldrete score of at least 9.

### 2.5. Outcomes

The primary outcome was participant-level respiratory depression from induction until completion of the procedure. Respiratory depression was defined as any episode of respiratory rate below 10 breaths/min or peripheral oxygen saturation below 90% lasting longer than 1 min. The blinded observer assessed the continuously displayed monitor values. The composite outcome was recorded directly once per child, irrespective of the number of qualifying episodes.

Secondary outcomes were sedation success, cardiovascular depression, number of supplemental propofol boluses, procedure duration, time to eye opening, and other adverse events. Sedation success required completion of the endoscopic procedure without more than five supplemental sedative administrations in any 15 min interval. Cardiovascular depression was defined as systolic blood pressure below 70 mmHg plus twice the child’s age in years or heart rate below 60 beats/min. Procedure duration extended from successful gastroscope insertion to withdrawal of the colonoscope. Time to eye opening extended from cessation of anesthetic administration to eye opening. Other prespecified adverse events included intraoperative awareness, delirium, nausea, vomiting, and allergic reactions. All adverse events were assessed from induction through discharge from the post-anesthesia care unit.

### 2.6. Sample Size

Preliminary estimates for respiratory depression were 50% in P, 10% in R1, and 30% in R2. The sample size was calculated using PASS 2021 (NCSS, Kaysville, UT, USA), with a two-sided alpha of 0.05, 80% power, equal allocation, and comparison across the three groups; the estimated requirement was 32 children per group. Allowing for up to 20% attrition, the planned enrollment was 120.

### 2.7. Statistical Analysis

The primary analysis included all randomized children in their assigned groups. Continuous baseline characteristics are reported as mean (standard deviation), and categorical characteristics as count (percentage). Baseline significance tests were not performed because allocation was randomized.

The three-group primary outcome comparison used Pearson’s chi-square test. For R1-versus-P and R2-versus-P, absolute risk differences were calculated with Newcombe 95% CIs and risk ratios with score 95% CIs. The two primary pairwise *p* values were adjusted with the Holm method. The direct R1-versus-R2 comparison was exploratory and was not included in the multiplicity adjustment. Modified Poisson regression with robust standard errors estimated risk ratios adjusted for age and sex. Logistic regression using the same covariates was performed as a supplementary analysis. An age- and sex-adjusted linear model with heteroskedasticity-consistent standard errors was used as a supplementary analysis of time to eye opening. Models did not adjust for procedure duration or supplemental medication because these variables occurred after randomization.

Cardiovascular depression and the proportion requiring no supplemental propofol were compared with chi-square tests. Distribution and variance checks for time to eye opening and procedure duration are presented in Table S2. Supplemental propofol counts, procedure duration, and time to eye opening were non-normally distributed and were compared across groups with the Kruskal–Wallis test. Pairwise comparisons used Mann–Whitney tests. Holm correction was applied within each outcome to the two comparisons with P; R1-versus-R2 remained exploratory. No multiplicity adjustment was applied across distinct secondary outcomes, which were interpreted as exploratory.

Analyses were performed with Python 3.12, scipy 1.18.0, and statsmodels 0.14.6. Figures were generated with matplotlib 3.11.0. All tests were two-sided, and *p* < 0.05 was the threshold for statistical significance unless a multiplicity-adjusted *p* value was specified.

## 3. Results

### 3.1. Participant Flow and Baseline Characteristics

**Figure 1:**
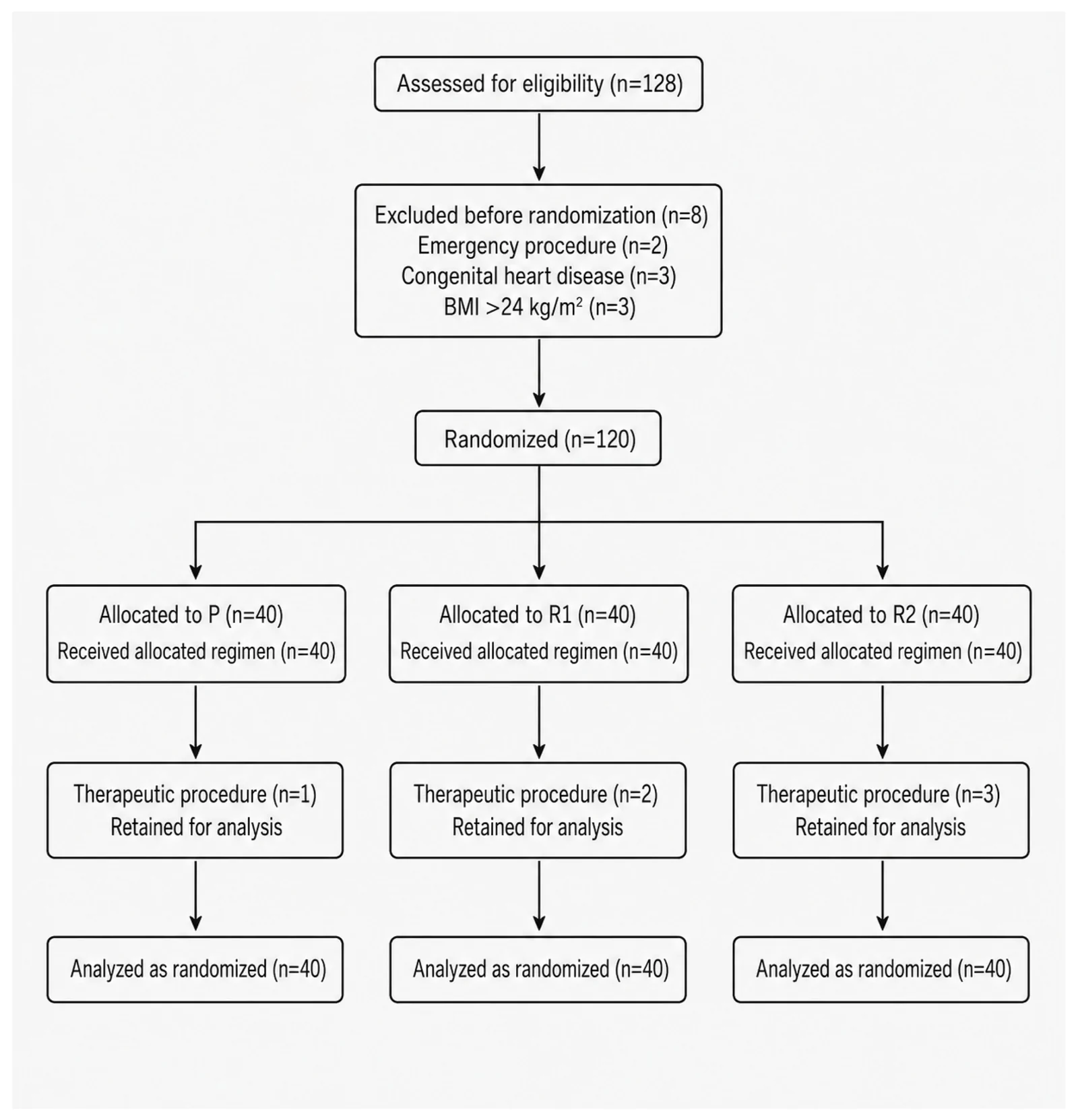
Participant flow through the randomized trial.

Of 128 children assessed, 120 were randomized and analyzed in their assigned groups (40 per group). Six procedures changed to therapeutic endoscopy after randomization, but these children remained in the primary analysis (Figure 1). The mean age was 9.6 years, 50.8% were male, and baseline characteristics are shown in Table 1.

### 3.2. Primary Outcome

**Figure 2:**
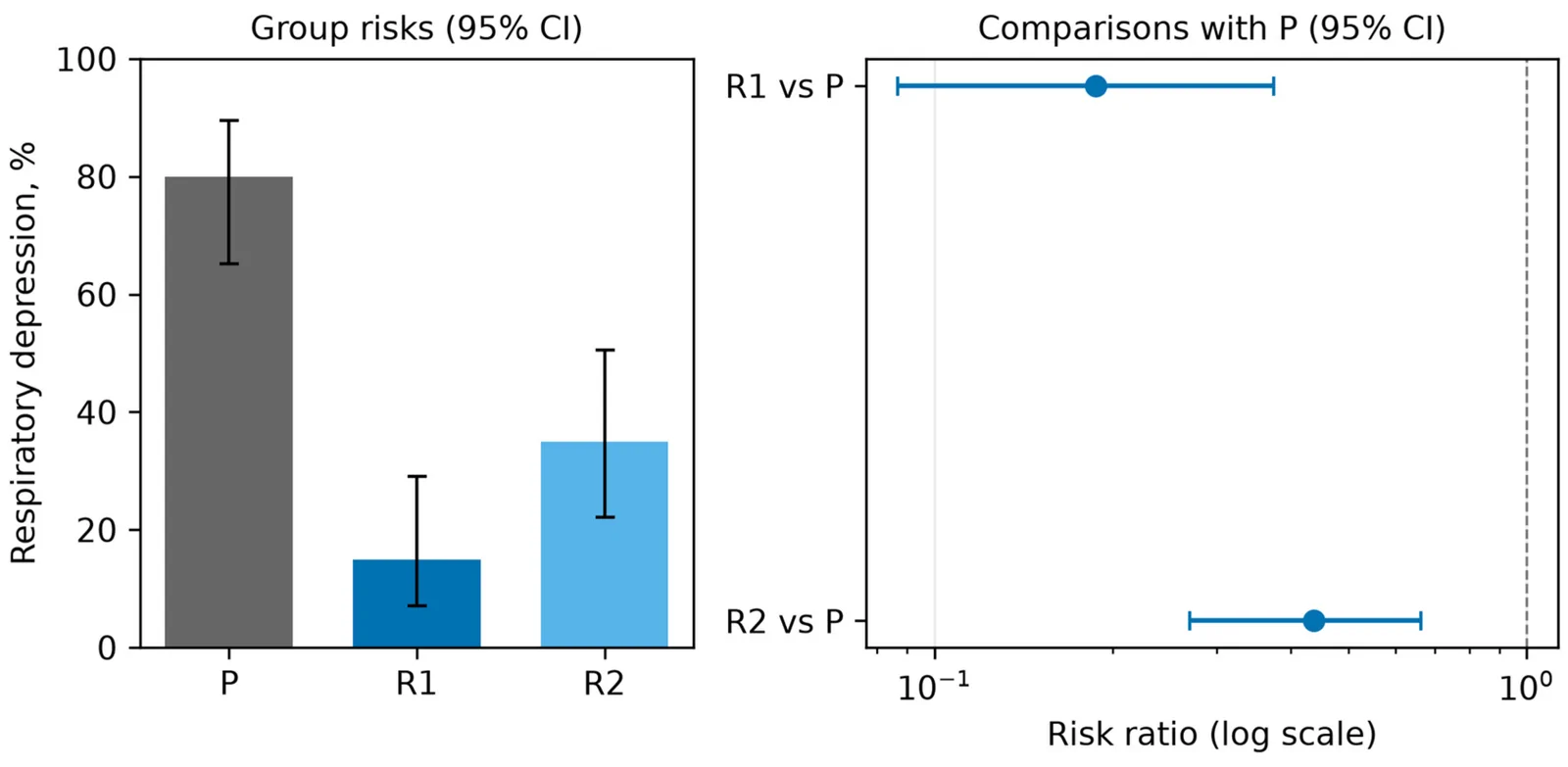
Respiratory depression risks and comparisons with the propofol regimen.

Respiratory depression occurred in 32/40 (80.0%) children in P, 6/40 (15.0%) in R1, and 14/40 (35.0%) in R2 (overall *p* < 0.001; Table 2 and Figure 2). Compared with P, the risk difference was −65.0 percentage points for R1 (95% CI, −77.4 to −44.6; risk ratio, 0.19, 95% CI, 0.09–0.37) and −45.0 percentage points for R2 (95% CI, −61.0 to −23.6; risk ratio, 0.44, 95% CI, 0.27–0.66); both Holm-adjusted *p* values were <0.001.

Age- and sex-adjusted risk ratios were 0.18 for R1-versus-P (95% CI, 0.09–0.38) and 0.42 for R2-versus-P (95% CI, 0.27–0.65); both Holm-adjusted *p* values were <0.001. The 114-participant sensitivity analysis gave similar estimates. Detailed sensitivity estimates, respiratory outcome components, and supplementary adjusted models are reported in Tables S1, S3 and S5. Airway interventions were not prospectively recorded and therefore cannot be reliably reported.

### 3.3. Secondary Outcomes

Cardiovascular depression did not differ significantly among P, R1, and R2 (27.5%, 15.0%, and 32.5%, respectively; *p* = 0.177). Sedation success was 40/40 (100%) in P, R1, and R2; because no sedation failure occurred, a comparative *p* value was not estimable. No intraoperative awareness, delirium, nausea, vomiting, or allergic reaction was recorded in any group through discharge from the post-anesthesia care unit (Table 3).

No supplemental propofol was required in 15.0% of P, 55.0% of R1, and 67.5% of R2 (*p* < 0.001). Mean time to eye opening was 12.7 min in P, 11.2 min in R1, and 16.9 min in R2. Compared with P, the mean difference was −1.45 min for R1 (95% bootstrap CI, −2.98 to −0.02; Holm-adjusted *p* = 0.026) and 4.30 min for R2 (95% bootstrap CI, 2.60–5.95; Holm-adjusted *p* < 0.001). In the exploratory R1-versus-R2 comparison, the mean difference was −5.75 min (95% bootstrap CI, −7.20 to −4.28; unadjusted *p* < 0.001). In the age- and sex-adjusted supplementary model, the R1-versus-P estimate was −1.48 min (95% CI, −3.01 to 0.06; Holm-adjusted *p* = 0.059), whereas the R2-versus-P estimate was 4.27 min (95% CI, 2.50–6.03; Holm-adjusted *p* < 0.001). Procedure duration did not differ among groups (*p* = 0.484). Pairwise secondary comparisons and adjusted models are reported in Tables S4 and S5.

## 4. Discussion

In this prospective, randomized three-arm trial of 120 school-aged children undergoing bidirectional endoscopy, both remimazolam–propofol regimens substantially reduced the primary composite respiratory depression outcome compared with the propofol 3 mg/kg regimen. The incidence was lowest with remimazolam 0.2 mg/kg plus propofol 1 mg/kg (15.0%), followed by remimazolam 0.4 mg/kg plus propofol 1 mg/kg (35.0%), versus 80.0% with the propofol regimen. Both combination regimens also reduced the need for supplemental propofol without increasing procedure duration or cardiovascular depression. Recovery differed between doses: the 0.4 mg/kg regimen prolonged time to eye opening, whereas the 0.2 mg/kg regimen showed recovery similar to propofol and was faster than the 0.4 mg/kg regimen in the exploratory direct comparison. These results support the primary hypothesis and identify the lower-dose combination as a promising regimen for further evaluation.

The 80.0% incidence in P reflects the prespecified monitor-defined composite rather than the frequency of severe complications requiring airway rescue. Among the 39 P-group children with complete component-level data, 19 (48.7%) had isolated sustained respiratory rate reduction, 6 (15.4%) had isolated oxygen desaturation, and 6 (15.4%) met both components; any oxygen desaturation occurred in 12/39 (30.8%), compared with 2/38 (5.3%) in R1 and 6/37 (16.2%) in R2. The high composite rate was therefore driven mainly by sustained respiratory rate reduction. This endpoint captured both ventilation and oxygenation: during supplemental oxygen, a decline in peripheral oxygen saturation may lag impaired ventilation, whereas the >1 min threshold reduces classification of transient fluctuations or isolated monitor alarms. This rationale is consistent with pediatric sedation guidance recommending monitoring of both ventilation and oxygenation during deep sedation [24]. Because monitoring conditions and blinded assessment were identical across groups, the composite remains useful for relative physiologic comparison but should not be interpreted as the rate of severe respiratory complications.

A plausible explanation for the respiratory benefit is the reduction in the propofol induction dose when remimazolam was incorporated into the regimen. Propofol produces dose-dependent respiratory depression, whereas remimazolam is rapidly hydrolyzed by tissue esterases and has generally shown a more stable respiratory profile during procedural sedation [2,3,4]. Distributing the hypnotic requirement across the two agents may therefore have preserved spontaneous ventilation while maintaining adequate conditions for endoscope insertion. The substantially higher proportion of children who required no supplemental propofol in R1 and R2 supports the adequacy of both combination regimens. Because adding remimazolam and reducing propofol occurred together, the observed benefit is best interpreted as an effect of the complete induction strategy rather than an isolated pharmacologic effect of either drug.

The present findings are consistent with adult randomized trials in gastrointestinal endoscopy, which have generally reported fewer respiratory events with remimazolam-based sedation than with propofol-based sedation [5,6,7,8,9,10], as well as with systematic reviews and meta-analyses showing a similar direction of effect across endoscopic procedures [11,12,13,14,15,16,17]. Pediatric evidence remains less extensive but is also supportive. Chu et al. reported fewer respiratory events with remimazolam than with propofol when both were combined with low-dose esketamine for pediatric bidirectional endoscopy [18]. Other studies have demonstrated the feasibility of remimazolam for pediatric general anesthesia, magnetic resonance imaging, and gastrointestinal endoscopy [19,20,21]. The current trial extends this evidence by comparing two remimazolam–propofol induction regimens with a fixed-dose propofol regimen in the same bidirectional endoscopy setting. Differences in co-medication, depth of anesthesia, oxygen delivery, and respiratory event definitions should be considered when comparing effect sizes across studies [22].

No intraoperative awareness, delirium, nausea, vomiting, or allergic reactions were observed through discharge from the post-anesthesia care unit. The absence of these events may reflect the relatively healthy study population, the brief diagnostic procedures, low-dose sufentanil use, and standardized intravenous anesthesia. However, follow-up ended at discharge from the post-anesthesia care unit, and the trial was not powered to detect rare adverse events; therefore, the zero counts should be interpreted cautiously.

The dose-related pattern observed in this trial is clinically informative. Although both remimazolam regimens reduced respiratory depression relative to P, the exploratory R1-versus-R2 analysis favored R1 for both respiratory depression and time to eye opening. The longer recovery after 0.4 mg/kg may reflect a dose-dependent residual sedative effect, particularly when remimazolam is followed by propofol induction and maintenance. Conversely, 0.2 mg/kg provided a marked reduction in respiratory depression, reduced supplemental propofol requirements, and did not prolong recovery relative to P. This pattern suggests that increasing the remimazolam dose does not necessarily produce a more favorable overall recovery profile. However, the direct comparison between remimazolam doses was exploratory, and the trial was not specifically powered to establish an optimal dose. The higher respiratory event rate and longer recovery observed in R2 than in R1 require confirmation in a larger trial designed and powered for a direct dose comparison.

These findings are relevant to pediatric endoscopy, where an effective regimen must provide immobility and procedural tolerance while preserving spontaneous ventilation and allowing timely recovery. The absolute reductions in monitor-defined respiratory depression were large, and the reduced need for supplemental propofol may simplify anesthetic management during a procedure that involves both upper and lower endoscopy. Within the studied population and protocol, the 0.2 mg/kg remimazolam–propofol regimen appeared to provide the most favorable balance between respiratory stability and recovery. This observation should be viewed as a dose-selection signal rather than a definitive practice recommendation, and standard monitoring, oxygen supplementation, and readiness for airway support remain essential with any deep sedation regimen.

Important strengths of this study include its prospective randomized design, equal allocation to three parallel groups, blinded outcome assessment, standardized co-medication and propofol maintenance, prespecified rescue procedures, and analysis of all randomized children in their assigned groups. Reporting absolute and relative treatment effects with confidence intervals, together with adjusted and sensitivity analyses, further supports the consistency of the primary finding. The inclusion of two remimazolam doses also provides clinically useful information about the relationship between induction dose and recovery.

Several limitations should be considered. This was a single-center trial in relatively healthy children aged 6–12 years, so the findings may not generalize to younger children, patients with substantial comorbidity, or other procedures. The treating anesthesiologist could not be blinded, which may have influenced supplemental propofol administration. Because the propofol induction dose differed between P and the two combination groups, the study compares complete regimens and cannot separate the contribution of remimazolam from that of propofol dose reduction. The primary outcome was designed as a physiologic monitoring endpoint rather than a measure of airway rescue. Accordingly, the trial did not prospectively classify each qualifying event by intervention type or level, procedure interruption, or assisted ventilation. The observed between-regimen differences therefore establish a reduction in the prespecified monitor-defined composite but should not be interpreted as demonstrating an equivalent reduction in severe airway-rescue events. The direct dose comparison and secondary outcomes were exploratory. Future trials should record the timing and duration of respiratory episodes together with the corresponding intervention type and level, procedure interruption, and assisted ventilation. Nevertheless, the magnitude and consistency of the randomized primary comparison provide supportive evidence for remimazolam–propofol induction strategies in this setting.

## 5. Conclusions

In relatively healthy school-aged children undergoing diagnostic bidirectional endoscopy, remimazolam 0.2 or 0.4 mg/kg combined with propofol 1 mg/kg reduced monitor-defined respiratory depression compared with propofol 3 mg/kg. Both combination regimens also reduced supplemental propofol requirements, and the exploratory comparison indicated faster recovery with the 0.2 mg/kg than with the 0.4 mg/kg regimen. Within the conditions of this trial, the 0.2 mg/kg combination yielded a favorable balance between monitor-defined respiratory stability and recovery. Because the dose comparison was exploratory, this finding should be regarded as a dose-selection signal requiring confirmation rather than proof of an optimal dose or a definitive practice recommendation.

## Figures and Tables

**Table 1:** Baseline characteristics by randomized group.

Characteristic	P (n = 40)	R1 (n = 40)	R2 (n = 40)
Age, years	9.4 (1.7)	9.7 (1.9)	9.8 (1.9)
Body weight, kg	36.4 (7.2)	37.2 (10.3)	37.0 (10.2)
Height, cm	138.2 (11.3)	137.6 (19.0)	138.3 (18.6)
Body mass index, kg/m^2^	19.0 (2.8)	19.5 (2.6)	19.1 (2.2)
Male sex	20/40 (50.0%)	22/40 (55.0%)	19/40 (47.5%)
Indication for endoscopy			
Abdominal pain or diarrhea	14/40 (35.0%)	10/40 (25.0%)	15/40 (37.5%)
Nausea or vomiting	13/40 (32.5%)	18/40 (45.0%)	14/40 (35.0%)
Follow-up of chronic gastrointestinal disease	13/40 (32.5%)	12/40 (30.0%)	11/40 (27.5%)

Data are mean (standard deviation) or n/N (%). Baseline hypothesis tests were not performed. P, propofol 3 mg/kg; R1, remimazolam 0.2 mg/kg plus propofol 1 mg/kg; R2, remimazolam 0.4 mg/kg plus propofol 1 mg/kg.

**Table 2:** Primary respiratory depression outcome.

Contrast	Risk, First Group	Risk, Reference Group	Risk Difference, Percentage Points (95% CI)	Risk Ratio (95% CI)	*p* Value
R1 vs. P	6/40 (15.0%)	32/40 (80.0%)	−65.0 (−77.4 to −44.6)	0.19 (0.09 to 0.37)	<0.001
R2 vs. P	14/40 (35.0%)	32/40 (80.0%)	−45.0 (−61.0 to −23.6)	0.44 (0.27 to 0.66)	<0.001
R1 vs. R2 (exploratory)	6/40 (15.0%)	14/40 (35.0%)	−20.0 (−37.4 to −0.9)	0.43 (0.18 to 0.97)	0.040

CI, confidence interval. Risk difference CIs use the Newcombe method; risk ratio CIs use the score method. The R1-versus-P and R2-versus-P *p* values are Holm adjusted. The exploratory R1-versus-R2 *p* value is unadjusted.

**Table 3:** Secondary outcomes by randomized group.

Outcome	P (n = 40)	R1 (n = 40)	R2 (n = 40)	Overall P
Cardiovascular depression	11/40 (27.5%)	6/40 (15.0%)	13/40 (32.5%)	0.177
No supplemental propofol	6/40 (15.0%)	22/40 (55.0%)	27/40 (67.5%)	<0.001
Supplemental propofol administrations, median (IQR)	2.0 (1.0 to 3.0)	0.0 (0.0 to 1.0)	0.0 (0.0 to 1.0)	<0.001
Time to eye opening, min, mean (SD)	12.7 (3.8)	11.2 (2.9)	16.9 (3.8)	<0.001
Procedure duration, min, median (IQR)	35.5 (32.0 to 40.2)	35.0 (26.8 to 41.0)	37.0 (32.0 to 42.2)	0.484
Sedation success	40/40 (100%)	40/40 (100%)	40/40 (100%)	Not estimable
Intraoperative awareness	0/40 (0.0%)	0/40 (0.0%)	0/40 (0.0%)	Not estimable
Delirium	0/40 (0.0%)	0/40 (0.0%)	0/40 (0.0%)	Not estimable
Nausea	0/40 (0.0%)	0/40 (0.0%)	0/40 (0.0%)	Not estimable
Vomiting	0/40 (0.0%)	0/40 (0.0%)	0/40 (0.0%)	Not estimable
Allergic reactions	0/40 (0.0%)	0/40 (0.0%)	0/40 (0.0%)	Not estimable

Data are n/N (%), median (interquartile range), or mean (standard deviation), as indicated. Categorical outcomes were compared with chi-square tests; count and continuous outcomes were compared with Kruskal–Wallis tests.

## Data Availability

The data presented in this study are available on request from the corresponding author. The data are not publicly available due to privacy and ethical reasons.

## Supplementary Materials

The following supporting information can be downloaded at https://www.mdpi.com/article/10.3390/children13081056/s1. Table S1: Sensitivity analysis excluding six post-randomization therapeutic conversions; Table S2: Distribution and variance checks for continuous outcomes; Table S3: Respiratory depression components among children with component-level data; Table S4: Pairwise comparisons of selected secondary outcomes; Table S5: Supplementary age- and sex-adjusted models.

## Abbreviations

ASA, American Society of Anesthesiologists; CI, confidence interval; P, propofol 3 mg/kg group; R1, remimazolam 0.2 mg/kg plus propofol 1 mg/kg group; R2, remimazolam 0.4 mg/kg plus propofol 1 mg/kg group.

## References

[1] Bruni A., Barbara G., Vitello A., Marasco G., Maida M. (2025). Sedation in endoscopy: Current practices and future innovations. World J. Gastrointest. Endosc..

[2] Noor N., Legendre R., Cloutet A., Chitneni A., Varrassi G., Kaye A.D. (2021). A comprehensive review of remimazolam for sedation. Health Psychol. Res..

[3] Hu Q., Liu X., Wen C., Li D., Lei X. (2022). Remimazolam: An updated review of a new sedative and anaesthetic. Drug Des. Dev. Ther..

[4] Zhang H., Li H., Zhao S., Bao F. (2024). Remimazolam in general anesthesia: A comprehensive review of its applications and clinical efficacy. Drug Des. Dev. Ther..

[5] Chen S., Yuan T., Zhang J., Bai H., Tian M., Pan C., Bao H., Jin X., Ji F., Zhong T. (2021). Remimazolam tosilate in upper gastrointestinal endoscopy: A multicenter, randomized, non-inferiority, phase III trial. J. Gastroenterol. Hepatol..

[6] Lu K., Wei S., Ling W., Wei Y., Ran X., Huang H., Wang M., Wei N., Liao Y., Qin Z. (2022). Remimazolam versus propofol for deep sedation/anaesthesia in upper gastrointestinal endoscopy in elderly patients: A multicenter, randomized controlled trial. J. Clin. Pharm. Ther..

[7] Hu B., Jiang K., Shi W., Xiao S., Zhang S., Zhang Y., Zhou Y., Tan C., Tan S., Zou X. (2022). Effect of remimazolam tosilate on respiratory depression in elderly patients undergoing gastroscopy: A multicentered, prospective, and randomized study. Drug Des. Dev. Ther..

[8] Shi H., Zhang J., Hu Z., Hou Q., Hu Q., Dai Z., Zhou W., Qi D., Li Y., Wang Q. (2024). The efficacy and safety of remimazolam in painless colonoscopy: A prospective, randomized clinical trial. Front. Med..

[9] Wei A., Ma S., Dou Y., Wang X., Wu J., Zhou S., Deng Y., Liu X., Li D., Yang M. (2023). The safety and efficacy of remimazolam tosylate combined with propofol in upper gastrointestinal endoscopy: A multicenter, randomized clinical trial. PLoS ONE.

[10] Wang C., Gao Y., Li J., Zhang L., Li Q., Li Y., Lu Y., Sun J., Zhang Y., Cheng Y. (2023). Safety and effectiveness of the combination of remimazolam tosilate and propofol in gastroscopy: A multicenter, randomized controlled, single-blind clinical trial. Front. Pharmacol..

[11] Barbosa E.C., Espirito Santo P.A., Baraldo S., Meine G.C. (2024). Remimazolam versus propofol for sedation in gastrointestinal endoscopic procedures: A systematic review and meta-analysis. Br. J. Anaesth..

[12] An X., Shen T., Yin X., Xu J., Zhang Y., Wang T. (2024). The safety of remimazolam versus propofol in gastroscopic sedation: A meta-analysis. BMC Anesthesiol..

[13] Chang Y., Huang Y.-T., Chi K.-Y., Huang Y.-T. (2023). Remimazolam versus propofol for procedural sedation: A meta-analysis of randomized controlled trials. PeerJ.

[14] Xin Y., Lu P., Guan S., Si S., Sun R., Xia W., Xu H. (2025). Efficacy and safety of remimazolam in short endoscopic procedures: A systematic review and meta-analysis. Medicina.

[15] Zhou S., Yu S., Bi Y., Tian Z., Pan R., Yan T., Deng J., Xu A. (2025). The safety and efficacy of remimazolam, ciprofol, and propofol anesthesia in endoscopy: A systematic review and network meta-analysis. BMC Anesthesiol..

[16] Xing Y., Lang Z., Wang X., Liu J., Han X., Zhou J., Leng Y. (2025). Anesthetic efficacy with remimazolam compared with propofol: A systematic review and meta-analysis of randomized controlled trials. Int. J. Surg..

[17] Kim I.J., Choi G.J., Oh H.-C., Kang H. (2025). Comparison of remimazolam and propofol for sedation in endoscopic retrograde cholangiopancreatography: A systematic review and meta-analysis with trial sequential analysis. Dig. Endosc..

[18] Chu T., Zhou S., Wan Y., Liu Q., Xin Y., Tian Z., Yan T., Xu A. (2024). Comparison of remimazolam and propofol combined with low-dose esketamine for pediatric same-day painless bidirectional endoscopy: A randomized, controlled clinical trial. Front. Pharmacol..

[19] Fang Y., Zhong J.W., Szmuk P., Lyu Y., Xu Y., Qu S., Du Z., Shangguan W., Liu H. (2025). Safety and efficacy of remimazolam tosilate for general anaesthesia in paediatric patients undergoing elective surgery: A multicentre, randomised, single-blind, controlled trial. Anaesthesia.

[20] Shioji N., Everett T., Suzuki Y., Aoyama K. (2022). Pediatric sedation using dexmedetomidine and remimazolam for magnetic resonance imaging. J. Anesth..

[21] Chen W., Shi L., Bao W., Liu Y., Shi J. (2026). Optimal remimazolam dosage for pediatric painless gastrointestinal endoscopy: A randomized controlled trial comparing efficacy and safety of three doses combined with sufentanil and propofol. BMC Anesthesiol..

[22] Zhu M., Tang M. (2025). Remimazolam as a novel anesthetic in pediatric procedural sedation and anesthesia: A narrative review. Cureus.

[23] Hopewell S., Chan A.-W., Collins G.S., Hróbjartsson A., Moher D., Schulz K.F., Tunn R., Aggarwal R., Berkwits M., Berlin J.A. (2025). CONSORT 2025 statement: Updated guideline for reporting randomised trials. PLoS Med..

[24] Coté C.J., Wilson S., American Academy of Pediatrics, American Academy of Pediatric Dentistry (2019). Guidelines for Monitoring and Management of Pediatric Patients Before, During, and After Sedation for Diagnostic and Therapeutic Procedures. Pediatrics.

