# Supplementary material for: Remimazolam–Propofol and Propofol Induction Regimens for Pediatric Bidirectional Endoscopy: A Three-Arm Randomized Trial
Source: Children (Basel). 2026 Aug 8;13(8):1056. doi: 10.3390/children13081056 (PMC13511081; doi:10.3390/children13081056)
Supplement: Supplementary file 1 [file children-13-01056-s001.zip › children-4447389-supplementary.pdf]

children

MDPI

Supplementary Materials

Remimazolam-Propofol and Propofol Induction Regimens for Pediatric Bidirectional Endoscopy: A Three-Arm Randomized Trial

Table S1. Sensitivity analysis excluding six post-randomization therapeutic conversions.

Panel A. Group risks

Group	Events/N (%)	Risk (95% CI)
P	31/39 (79.5%)	79.5% (64.5-89.2)
R1	5/38 (13.2%)	13.2% (5.8-27.3)
R2	12/37 (32.4%)	32.4% (19.6-48.5)

Panel B. Pairwise contrasts

Contrast	Risk difference, percentage points (95% CI)	Risk ratio (95% CI)	*P* value
R1 vs P	-66.3 (-78.6 to -45.7)	0.17 (0.07 to 0.35)	<0.001
R2 vs P	-47.1 (-63.1 to -25.0)	0.41 (0.24 to 0.64)	<0.001
R1 vs R2 (exploratory)	-19.3 (-37.0 to -0.2)	0.41 (0.16 to 0.99)	0.048

CI, confidence interval. The overall three-group chi-square *P* value was <0.001. The R1-versus-P and R2-versus-P *P* values are Holm adjusted; the exploratory R1-versus-R2 *P* value is unadjusted.

Table S2. Distribution and variance checks for continuous outcomes.

Outcome	Check	Group	Statistic	*P* value
Time to eye opening	Shapiro-Wilk	P	0.871	<0.001
Time to eye opening	Shapiro-Wilk	R1	0.896	0.001
Time to eye opening	Shapiro-Wilk	R2	0.939	0.032
Time to eye opening	Levene (median centered)	All groups	0.838	0.435
Procedure duration	Shapiro-Wilk	P	0.925	0.011
Procedure duration	Shapiro-Wilk	R1	0.856	<0.001
Procedure duration	Shapiro-Wilk	R2	0.808	<0.001
Procedure duration	Levene (median centered)	All groups	0.845	0.432

Shapiro-Wilk tests indicated non-normal distributions in each group; therefore, Kruskal-Wallis and Mann-Whitney tests were used for group comparisons. Levene tests were centered at the median.

Table S3. Respiratory depression components among children with component-level data.

Component	P (n = 39)	R1 (n = 38)	R2 (n = 37)
Bradypnea without oxygen desaturation	19/39 (48.7%)	3/38 (7.9%)	6/37 (16.2%)
Oxygen desaturation without bradypnea	6/39 (15.4%)	0/38 (0.0%)	2/37 (5.4%)
Concurrent bradypnea and oxygen desaturation	6/39 (15.4%)	2/38 (5.3%)	4/37 (10.8%)

Data are n/N (%). Component-level respiratory-rate and oxygen-saturation data were unavailable for six children whose procedures changed to therapeutic endoscopy after randomization.

Table S4. Pairwise comparisons of selected secondary outcomes.

Outcome	Contrast	Effect estimate	*P* value
Time to eye opening	R1 vs P	Mean difference -1.45 min (95% bootstrap CI, -2.97 to -0.02)	0.026
Time to eye opening	R2 vs P	Mean difference 4.30 min (95% bootstrap CI, 2.60 to 5.95)	<0.001
Time to eye opening	R1 vs R2 (exploratory)	Mean difference -5.75 min (95% bootstrap CI, -7.20 to -4.28)	<0.001
Supplemental propofol administrations	R1 vs P	Not estimated	<0.001
Supplemental propofol administrations	R2 vs P	Not estimated	<0.001
Supplemental propofol administrations	R1 vs R2 (exploratory)	Not estimated	0.262

CI, confidence interval. For each outcome, *P* values for R1 versus P and R2 versus P are Holm adjusted; R1 versus R2 is exploratory and unadjusted. No multiplicity adjustment was applied across distinct secondary outcomes. Mean-difference CIs were obtained from 10,000 bootstrap resamples; *P* values are from Mann-Whitney tests.

Table S5. Supplementary age- and sex-adjusted models.

Outcome	Model	Contrast	Effect	Estimate (95% CI)	*P* value
Respiratory depression	Modified Poisson with robust SE; adjusted for age and sex	R1 vs P	Adjusted risk ratio	0.18 (0.09 to 0.38)	<0.001
Respiratory depression	Modified Poisson with robust SE; adjusted for age and sex	R2 vs P	Adjusted risk ratio	0.42 (0.27 to 0.65)	<0.001
Respiratory depression	Logistic regression; adjusted for age and sex	R1 vs P	Adjusted odds ratio	0.03 (0.01 to 0.12)	<0.001
Respiratory depression	Logistic regression; adjusted for age and sex	R2 vs P	Adjusted odds ratio	0.11 (0.04 to 0.31)	<0.001
Time to eye opening, min	Linear regression with HC3 SE; adjusted for age and sex	R1 vs P	Adjusted mean difference	-1.48 (-3.01 to 0.06)	0.059
Time to eye opening, min	Linear regression with HC3 SE; adjusted for age and sex	R2 vs P	Adjusted mean difference	4.27 (2.50 to 6.03)	<0.001

CI, confidence interval. Models were adjusted for age and sex. Within each model, the two comparisons with P were Holm adjusted. These models were treated as post hoc exploratory analyses because prespecification could not be confirmed from the archived files.
